# Global and regional burden, temporal trends, and projections of chronic pain from 1990 to 2032, and its association with cardiovascular diseases: analyses based on global burden of diseases study 2021

**DOI:** 10.3389/fpubh.2025.1636949

**Published:** 2025-11-14

**Authors:** Yan-Li Zhang, Xiao-Chen Wu, Xiao-Yan Chen, Feng Gao, Jian Wang

**Affiliations:** Department of Cardiovascular Surgery, The General Hospital of Western Theater Command, College of Medicine, Southwest Jiaotong University, Chengdu, Sichuan, China

**Keywords:** chronic pain, socio-demographic index, global burden of diseases study, age-sex subgroups, cardiovascular disease

## Abstract

**Introduction:**

Chronic pain is a major global health problem that significantly affects quality of life and increases the risk of cardiovascular diseases.

**Methods:**

Using the Global Burden of Disease (GBD) 2021 data, this study analyzed temporal trends in chronic pain across 204 countries and territories from 1990 to 2021. We further examined the influence of the Socio-demographic Index (SDI), explored age- and sex-specific patterns, and projected the future burden of chronic pain through 2032. Cardiovascular diseases data were also analyzed for correlations with chronic pain.

**Results:**

Results showed a significant positive association between SDI and age-standardized prevalence rate (ASPR), with higher burdens in more developed regions, especially for cancer- and arthritis-related pain. Apart from headaches, most types of chronic pain—including low back pain, neck pain, osteoarthritis-related pain, and rheumatoid arthritis-related pain—were more prevalent in older adults. Females were generally more affected by musculoskeletal and arthritis-related pain, while males showed higher rates of gout- and pancreatitis-related pain. Projections suggest that the prevalence of rheumatoid arthritis and other musculoskeletal pain will continue to rise, whereas gout-, back-, neck-, and pancreatitis-related pain are expected to decline. Notably, chronic pain showed significant positive correlations with several cardiovascular diseases, including ischemic heart disease and stroke.

**Discussion:**

The global burden of chronic pain remains substantial and unevenly distributed by sex, age, and SDI level. The observed association between chronic pain and cardiovascular diseases highlights the need for integrated management strategies targeting both conditions.

## Introduction

Chronic pain is a complex and multifactorial health condition characterized by persistent or recurrent pain that typically lasts longer than 3 months and represents one of the leading causes of disability worldwide (1, 2). Unlike acute pain, which serves as a protective mechanism of the body, chronic pain often persists beyond the normal tissue healing process and gradually evolves into an independent pathological condition. It exerts profound impacts on physical, psychological, and social health outcomes, significantly impairing patients’ quality of life, psychological wellbeing, and functional status (3–6). Epidemiological evidence suggests that depression and anxiety frequently co-occur with chronic pain, disproportionately affecting women, younger populations, and fibromyalgia patients (7, 8). Chronic pain is closely associated with increased disability rates, reduced work productivity, and a substantial socioeconomic burden, particularly pronounced in aging populations and resource-limited settings (9–11).

In recent years, the global health burden of chronic pain has garnered increasing attention. However, its prevalence, determinants, and health outcomes vary markedly across regions and populations. Factors such as socioeconomic development, access to healthcare services, demographic characteristics (e.g., age and gender), and comorbidities contribute to the significant heterogeneity in the epidemiological features of chronic pain. Evidence suggests that individuals with lower socioeconomic status are more susceptible to chronic pain, partly due to limited access to healthcare, unstable employment, and insufficient insurance coverage (12). Moreover, race and socioeconomic factors further influence disability outcomes associated with chronic pain. Data indicate that the prevalence of high-impact chronic pain among U. S. adults over age 50 in the lowest wealth quartile was 17.1%, significantly higher than the 5.6% found in the highest wealth quartile (13).

Despite growing recognition of chronic pain as a global health concern, temporal trends and geographic disparities remain insufficiently characterized. Moreover, few studies have systematically explored its associations with chronic pain and cardiovascular diseases on a global scale. To address these gaps, we utilized the GBD 2021 database to (1) analyze global, regional, and national trends in chronic pain from 1990 to 2021; (2) evaluate the effects of socio-demographic factors (SDI, age, and sex) on chronic pain patterns; and (3) investigate the relationship between chronic pain burden and major cardiovascular outcomes. The GBD framework provides standardized methodologies for cross-country comparisons, as further discussed in the study strengths section.

A recent research using GBD 2019 data offered valuable insights into the global epidemiology of chronic pain. However, it was constrained by outdated data and limited exploration of comorbidities. Consequently, we leverage updated GBD 2021 data with improved modeling frameworks, refined case definitions, and extended temporal coverage, providing a more current and comprehensive assessment of the global burden of chronic pain. We further examined disparities across socio-demographic strata and projected the global burden of chronic pain from 2022 to 2032 to evaluate its future impact on healthcare systems. In addition, given the potential shared pathophysiological mechanisms, risk profiles, and frequent comorbidity between chronic pain and cardiovascular diseases, we also explored their potential associations. Overall, this comprehensive analysis aims to generate evidence to inform targeted, context-specific prevention and intervention strategies and support global efforts in pain management and public health improvement.

## Methods

### Study overview

In the GBD 2021 study, the database included cross-sectional data from the Global Health Data Exchange. This dataset includes the global burden of 369 diseases and injuries, as well as 87 risk factors, across 21 regions and 204 countries from 1990 to 2021. These data were comprehensively evaluated to offer a detailed landscape of global health trends. The GBD regions consist of geographically proximate and epidemiologically similar countries and territories, which could be categorized into seven super-regions: Central and Eastern Europe and Central Asia, high-income regions, Latin America and the Caribbean, North Africa and the Middle East, South Asia, Southeast Asia, East Asia, Oceania, and sub-Saharan Africa (14).^1^ Additionally, we calculated the SDI for each country, which is a composite indicator reflecting the social and economic conditions influencing health outcomes in each region. Specifically, the SDI is a 0-to-1 geometric mean of three components, including the total fertility rate among women under 25 years of age, the mean years of schooling for individuals aged 15 and older, and the per capita income distributional lag index (2). Based on this scale, the SDI is divided into five categories: low, low-middle, middle, high-middle, and high (15). The criteria for each of these five SDI categories are detailed in Supplementary Table S1. We collected 11 chronic pain and 10 cardiovascular diseases from the dataset, including males and females in 16 age groups.

### Definition

Chronic pain was defined as pain persisting for at least 3 months. Data were obtained from the Global Health Data Exchange corresponding to GBD 2021. According to the International Classification of Diseases, 10th Revision (ICD-10), chronic pain encompassed multiple categories, including headache disorders (e.g., migraine, tension-type headache), musculoskeletal disorders (e.g., low back pain, neck pain, osteoarthritis, rheumatoid arthritis, gout), and other pain-related conditions such as pancreatitis, herpes zoster, and tumors. The detailed ICD-10 codes for these conditions are provided in Supplementary Table S1.

### Age and sex groups

Populations were stratified by sex and 5-year age intervals (0–4 to 80 years and older). Age-standardized rates for prevalence, incidence, and years lived with disability were calculated using the GBD world standard population.

### Statistical analyses

R software (version 4.4.2) was used for statistical analyses and data visualizations. Descriptive analyses were conducted to characterize the global and regional burden of chronic pain, comparing age-standardized prevalence rates (ASPRs) across age groups, sexes, and regions. World map data were merged with age-standardized prevalence rate (ASPR) and Estimated Annual Percentage Change (EAPC) metrics, and spatial analyses were conducted using a Queen’s contiguity matrix, which captures both edge- and corner-sharing relationships between neighboring countries and provides a more comprehensive representation of geographic proximity than the Rook’s case matrix. The results were visualized with the “ggplot2” package. Associations between ASPR and SDI were examined using Spearman’s correlation, and inequality was assessed using the slope index of inequality and the concentration index. Temporal trends were analyzed using an autoregressive integrated moving average (ARIMA) model, with optimal models selected based on the Akaike and Bayesian Information Criteria through the “forecast” package to project 10-year prevalence trends. Finally, cardiovascular diseases data from the GBD 2021 database were analyzed to explore correlations between chronic pain and cardiovascular diseases burden from 1990 to 2021.

## Results

### Global trends

**Figure 1:**
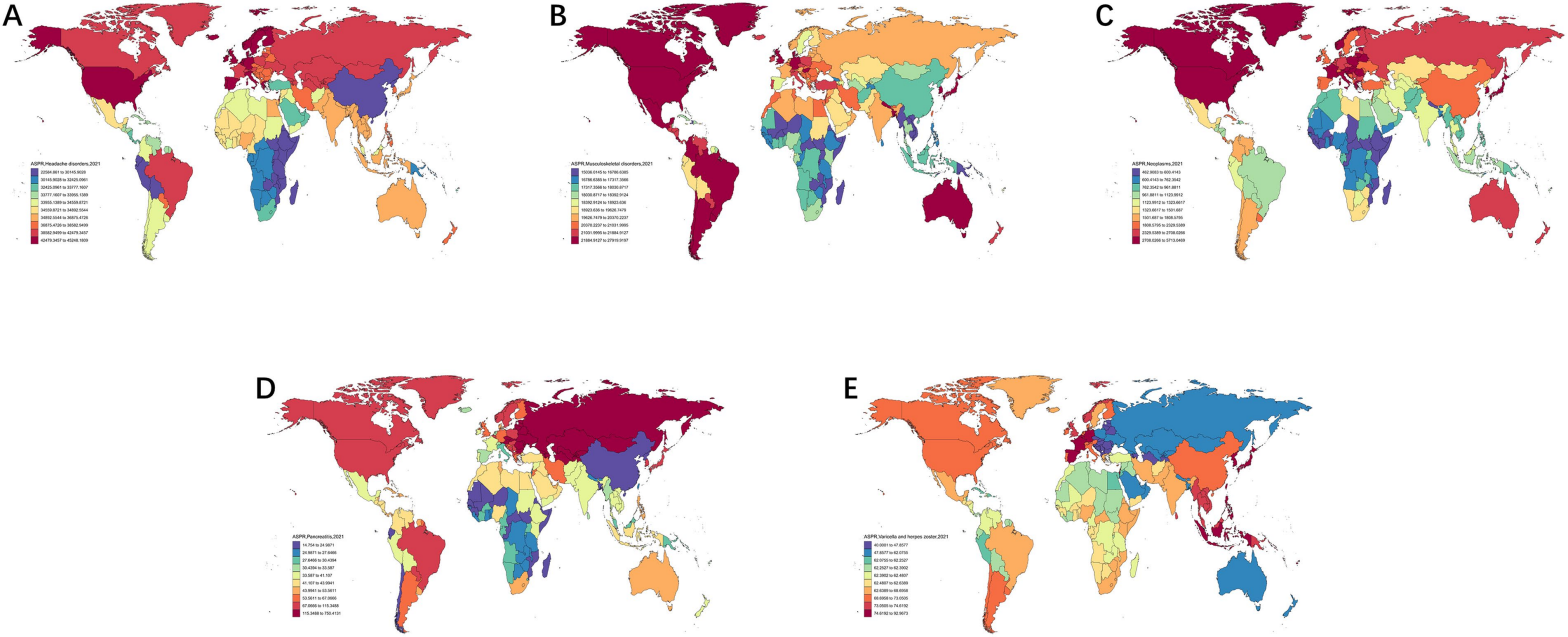
Distribution of ASPR for headache disorders **(A)**, musculoskeletal disorders **(B)**, neoplasms **(C)**, pancreatitis **(D)** and herpes zoster **(E)** in 204 countries (regions) worldwide in 2021.

To investigate the spatio-temporal distribution and trends of chronic pain burden across 204 countries/regions globally, data from the GBD 2021 database (1990–2021) were extracted. World map datasets were compiled and merged with ASPR of five chronic pain conditions (headache disorders, musculoskeletal disorders, tumours, pancreatitis, and herpes zoster) in R software. A queen’s case adjacency matrix was constructed for spatial autocorrelation analysis, with findings visualized using maps generated by the “ggplot2” package. These maps vividly illustrate the global distribution of chronic pain ASPR in 1990 (Supplementary Figure S1) and 2021 (Figure 1), presented sequentially from left to right for each condition.

As shown in Figure 1, both in 1990 and 2021, high-income regions such as North America, Japan, Korea, Europe, and Australia demonstrated higher ASPR for most chronic pain conditions, particularly headache and musculoskeletal disorders. However, low-income regions in sub-Saharan Africa generally suggested lower disease burdens. However, China showed differences. Specifically, while maintaining relatively high ASPR for tumour-related pain and herpes zoster, the country showed lower burdens for headache disorders, musculoskeletal disorders, and pancreatitis compared to developed nations.

### Impact of SDIs on the disease burden

**Figure 2:**
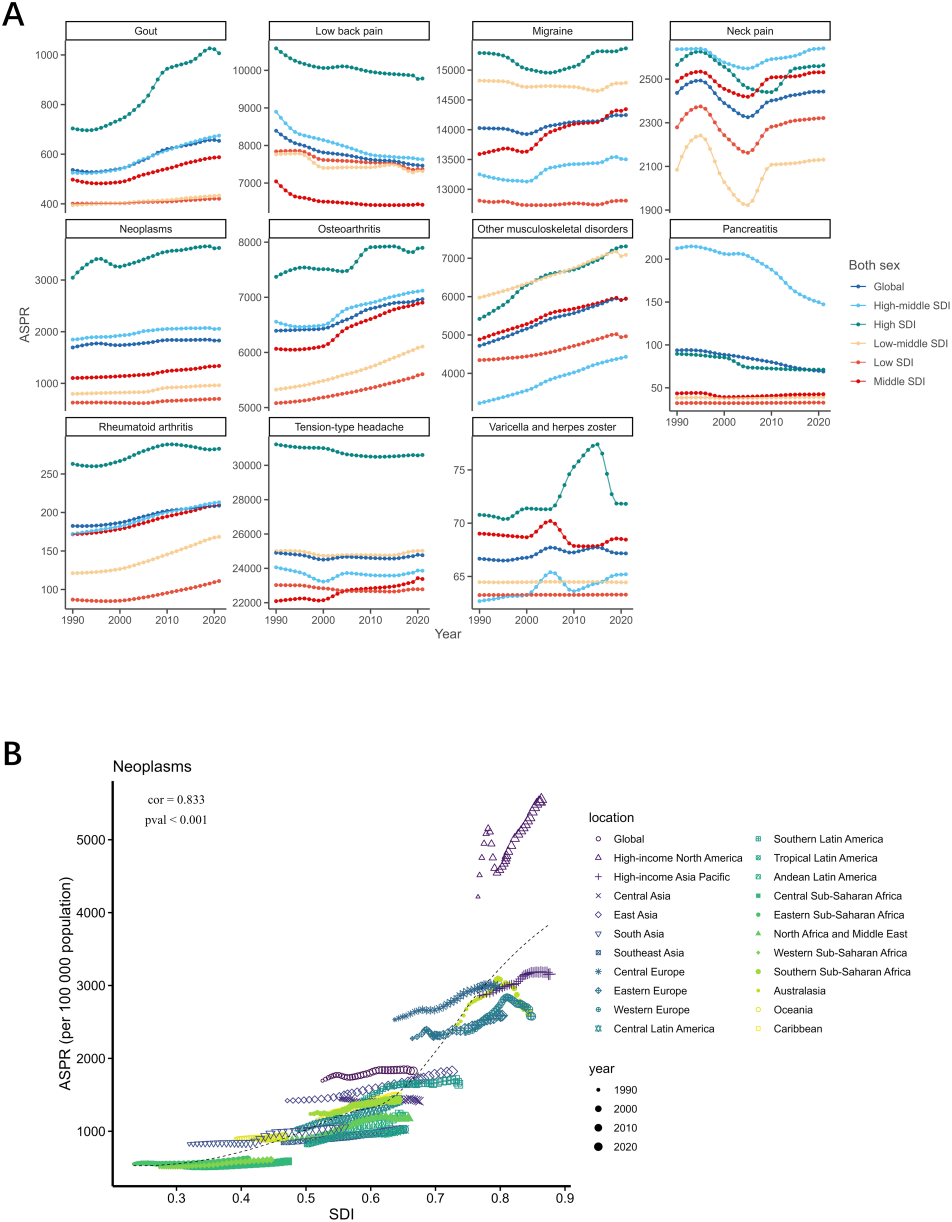
Trends in prevalence of chronic pain in relation to SDI, 1990–2021. **(A)** shows a line plot of different chronic pain subtypes with SDI, in which different line colours represent different SDI levels. The horizontal coordinate represents the year, and the vertical coordinate represents ASPR level; **(B)** shows a scatterplot of the correlation between tumour ASPR levels and different SDI regions, in which the colours and shapes of the scattered dots represent the different regions, and the sizes of the three dots represent the year.

Figure 2A illustrates the temporal trends of ASPR across five SDI quintiles (1990–2021). Notably, high-SDI and high-middle SDI regions demonstrated significant upward trends in chronic pain ASPR. However, stable prevalence patterns were observed in low-SDI, low-middle SDI, and middle-SDI regions. Figure 2B (Supplementary Figure S2) further reveals regional disparities across 21 GBD super-regions, suggesting tumour-related pain as the strongest correlate with SDI (Cor = 0.833, *p* < 0.001). This positive association revealed that higher SDI regions are subjected to greater tumour burdens, particularly in such countries as high-income North America. All other subtypes of chronic pain diseases, except for herpes zoster, are significantly positively correlated with the SDI.

A show a line plot of different chronic pain subtypes with SDI, in which different line colours represent different SDI levels. The horizontal coordinate represents the year, and the vertical coordinate represents ASPR level; B shows a scatterplot of the correlation between tumour ASPR levels and different SDI regions, in which the colours and shapes of the scattered dots represent the different regions, and the sizes of the three dots represent the year.

**Figure 3:**
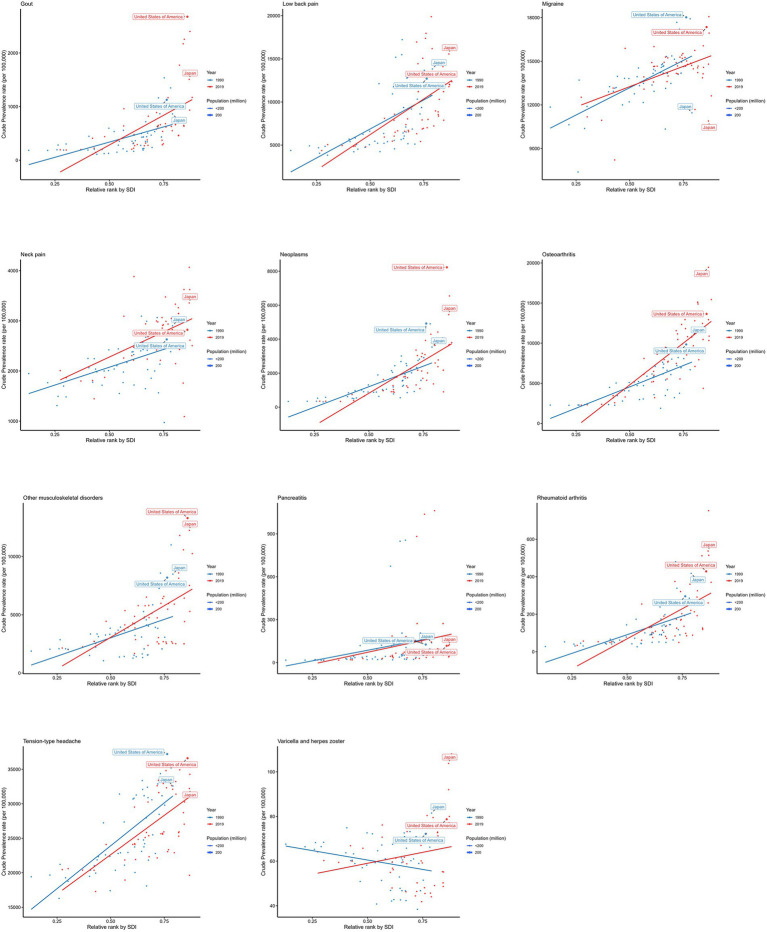
Health inequality regression curves for prevalence of chronic pain.

A cross-national inequality analysis was conducted based on the SDI. The 204 countries and regions were ranked according to their SDI values in ascending order. The cumulative population percentage of each country was calculated, and this value was combined with the country’s relative position to form the x-axis. Meanwhile, the prevalence rates were used as the y-axis. A robust regression model was then applied to fit the country-level data. The slope of the regression line, termed the slope index of inequality, assessing the health disparity between countries with the lowest and highest levels of socio-economic development. A smaller absolute value of the slope index of inequality indicates better health equity among countries. As shown in Figure 3, the increasing absolute slope over time suggests a worsening of global health equity.

Supplementary Figure S3 illustrates the concentration index, a metric to evaluate relative health equity. The concentration index (CI) is defined as twice the area between the diagonal equality line and the concentration curve. When the concentration curve lies above the diagonal, it represents negative inequality, suggesting that the disease burden affects countries with lower SDI values. Notably, when the curve lies below the diagonal, it indicates positive inequality, where higher-SDI countries are subjected to a greater burden. As shown in Supplementary Figure S3, chronic pain demonstrates positive inequality, with its prevalence concentrated in developed countries.

Our findings revealed that chronic pain burden has shifted toward high-SDI countries, indicating a form of pro-rich inequality supported by both the slope index of inequality (SII) and CI.

The horizontal coordinates in the altered figure represents the SDI levels, and the vertical coordinates represents the prevalence rates. The scatter dots represent different countries, the size of the dots represents the country’s population size, the red colour represents the status in 2019, and the blue colour represents the status in 1990.

### Disease burden of chronic pain in age-sex subgroups

**Figure 4:**
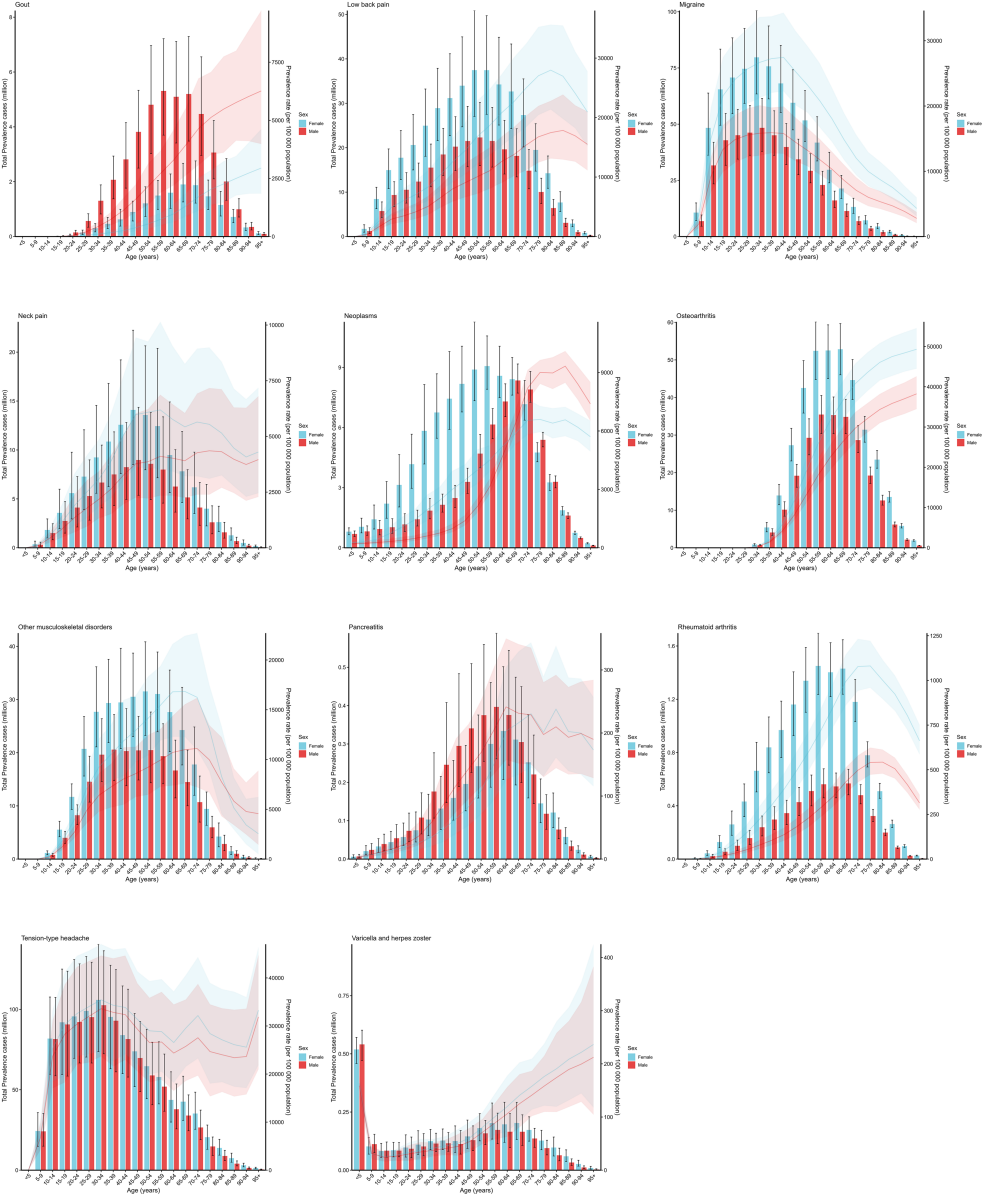
Age patterns of prevalence of chronic pain by age-sex, globally, 2021.

To better understand the distribution of chronic pain prevalence globally in 2021, patients were divided into age groups at five-year intervals, and gender distinctions were made within each age group. The findings are presented in Figure 4. The line chart in the figure illustrates the relationship between age, gender, and prevalence rates. It shows that, with the exception of migraine and tension-type headache, most subtypes of chronic pain are more prevalent among older individuals. The bar chart depicts the relationship between age, gender, and the number of cases. As indicated in the Figure 4, gout and pancreatitis are significantly more prevalent in females than in males. In contrast, lower back pain, migraine, neck pain, cancer, osteoarthritis, other musculoskeletal diseases, and rheumatoid arthritis are significantly more common in males than in females.

The horizontal coordinates of the graph represents age groupings, and the vertical coordinates represents the number of prevalences and prevalence rates.

### Trends in the burden of chronic pain disease

**Figure 5:**
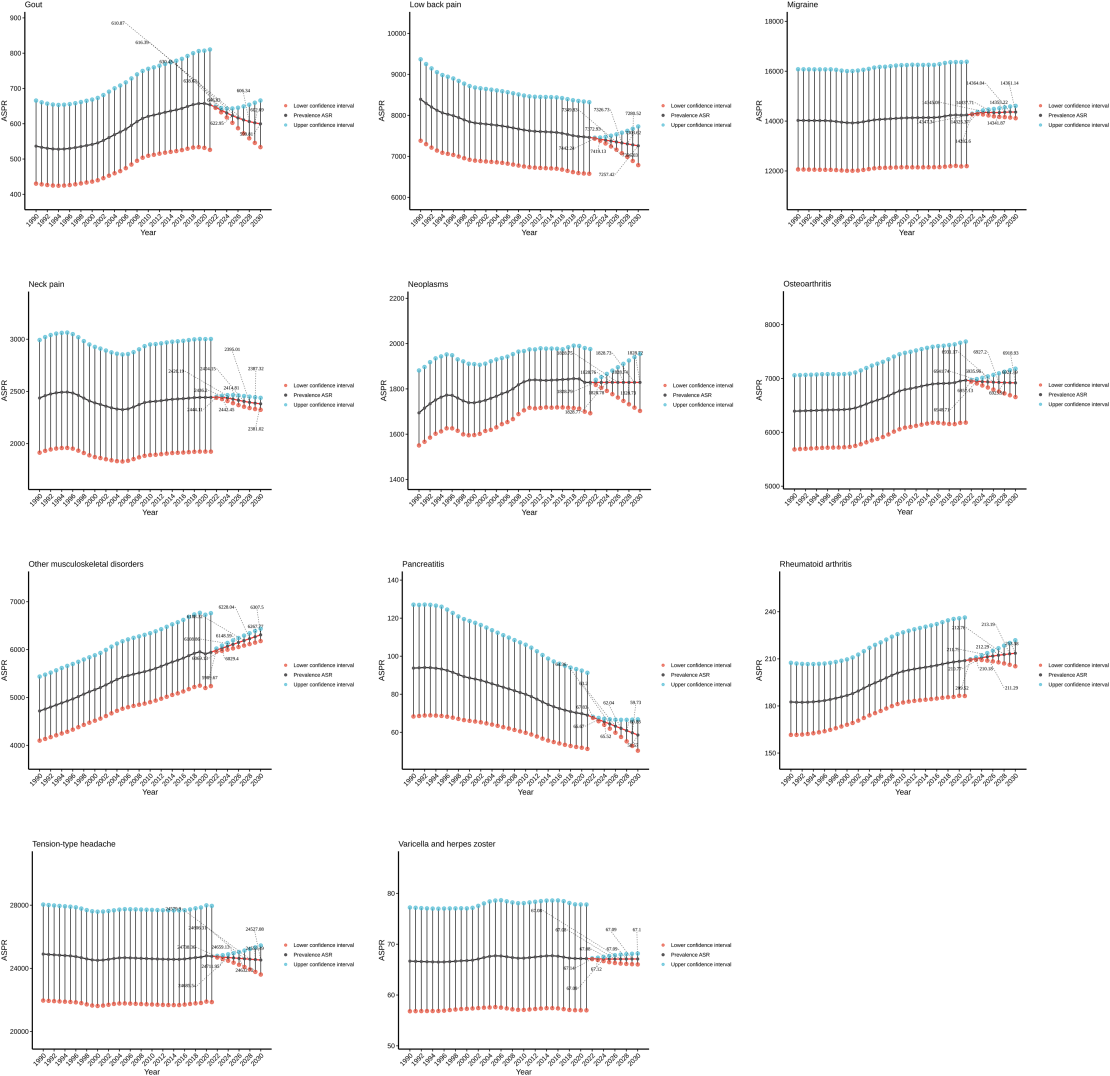
Predicted trends in the prevalence of chronic pain over the next decade.

To predict the prevalence of chronic pain globally over the next decade (2022–2032), an autoregressive integrated moving average model was constructed using data from 1990 to 2021, and a trend chart for the next ten years was plotted. The findings are shown in Figure 5. The prevalence of gout, lower back pain, neck pain, and pancreatitis is expected to show a significant deceasing trend, while the prevalence of rheumatoid arthritis and other musculoskeletal diseases is projected to increase significantly.

Red in this figure represents the lower interval of the 95% CI and blue represents the upper interval of the 95% concentration index.

### Correlation analysis between chronic pain and cardiovascular disease

**Figure 6:**
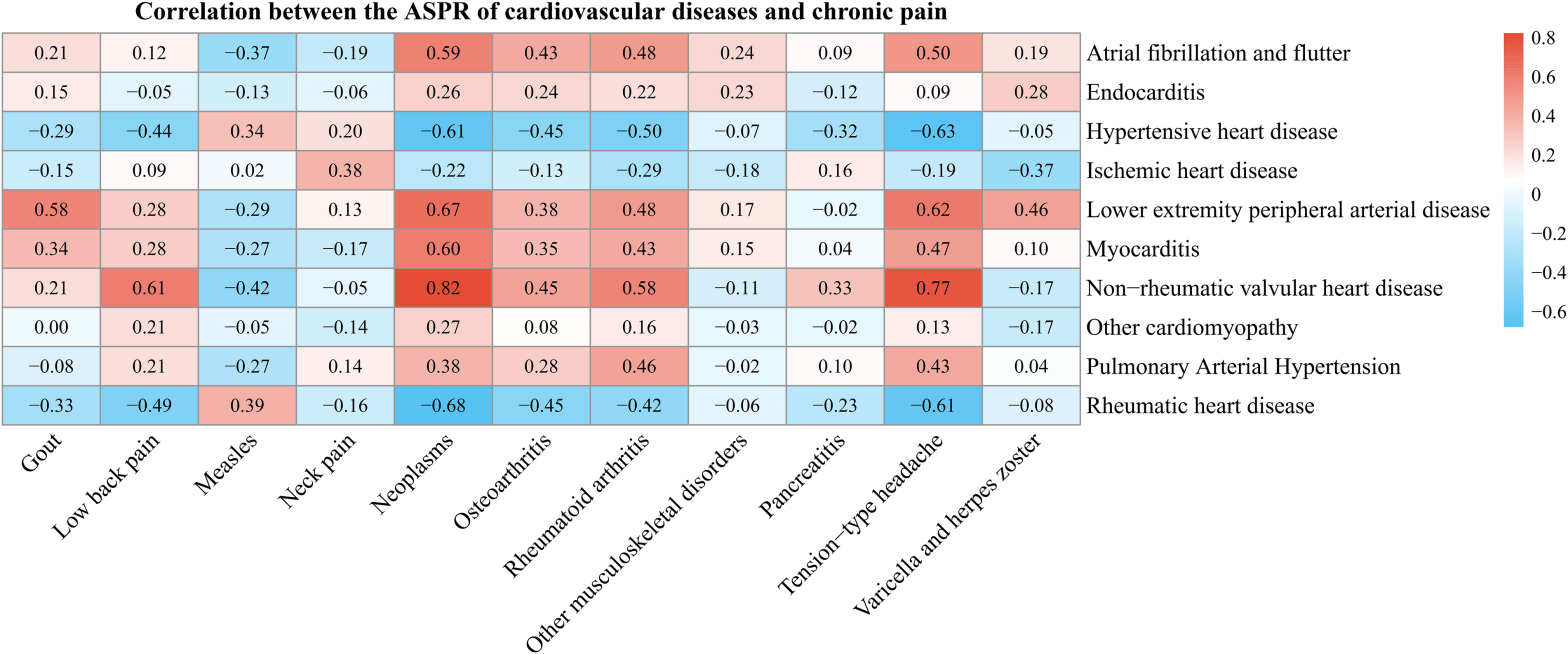
Heatmap of correlation between cardiovascular disease and prevalence of chronic pain.

To explore the potential association between cardiovascular diseases and chronic pain, a correlation analysis was conducted based on the prevalence of various cardiovascular diseases and chronic pain using data from the GBD 2021 database. As shown in Figure 6, certain types of chronic pain have a high correlation with the prevalence of cardiovascular diseases. For example, gout, lower back pain, neoplasm, osteoarthritis, rheumatoid arthritis, and tension-type headache are significantly positively correlated with conditions such as lower extremity peripheral artery disease, myocarditis, and non-rheumatic valvular heart disease (*p* < 0.05). However, lower back pain, neoplasm, osteoarthritis, non-rheumatic arthritis, and tension-type headache are significantly negatively correlated with hypertensive heart disease and rheumatic heart disease (*p* < 0.05).

## Discussion

This study systematically analyzed the epidemiological characteristics and disease burden of chronic pain worldwide, using data from GBD 2021. The analysis covered trends across countries, age groups, and sexes. Additionally, we examined the association between the SDI and chronic pain burden, and further explored the relationship between chronic pain and cardiovascular diseases.

Data from GBD 1990–2021 show a steady global rise in the ASPR of chronic pain, with marked regional disparities. Between 1990 and 2021, the ASPR gap between the highest and lowest burden countries expanded from 12084.21 to 12883.90, highlighting growing global polarization in chronic pain management. This may reflect the “Matthew effect” in healthcare (16): high-income countries, with better pain recognition and care systems, likely report rates closer to actual prevalence (17), while low- and middle-income countries may experience significant underdiagnosis and misdiagnosis due to limited access and awareness. To better account for this imbalance, we have further emphasized that underdiagnosis in low-SDI regions could lead to an underestimation of the true global burden, suggesting that disparities may be even larger than reported. This underrecognition might distort global projections, particularly in regions with limited healthcare infrastructure.

The estimated annual percentage change trend analysis further substantiates this disparity. Pakistan (+0.45%) and Burundi (−0.25%) exhibited the highest increase and the greatest decrease in ASPR, respectively, highlighting the dual challenges of accelerated population aging in high-income countries and limited healthcare resources in developing nations (18, 19). For instance, the persistently high prevalence in high-income countries such as Chile and the United States may be attributed to a surge in musculoskeletal disorders driven by sedentary lifestyles (20). In contrast, Eritrea’s low prevalence may be related to its youthful population structure (median age at 18.3 years) and regional variability in post-traumatic pain diagnostic criteria (1, 21, 22). These patterns underscore the substantial global imbalance in chronic pain burden distribution and emphasize the need for enhanced public health strategies and healthcare system strengthening in high-burden settings (16). From a biological perspective, the chronic nature of chronic pain may be partly driven by sustained sympathetic nervous system activation. Prolonged norepinephrine release can induce vasoconstriction, reduce heart rate variability, and elevate arterial pressure, potentially creating a vicious cycle that is associated with cardiovascular strain (23). Additionally, elevated levels of inflammatory cytokines such as IL-6 and TNF-*α* may activate both the hypothalamic–pituitary–adrenal axis and the renin-angiotensin-aldosterone system, further promoting hypertension and cardiovascular pathology (24). These mechanisms may partly explain the high prevalence of cardiovascular comorbidities observed in this study and suggest potential therapeutic targets for comprehensive chronic pain management (25).

Our findings reveal significant socioeconomic disparities in the global burden of chronic pain from 1990 to 2021. The ASPR of chronic pain increased overall in high and high-middle SDI regions, while remaining relatively stable in middle and low SDI areas. This trend suggests that as socioeconomic development progresses, both reported prevalence and actual burden of chronic pain rise, particularly in high-income countries. For instance, gout, a representative condition, shows a strong positive correlation with SDI (Cor = 0.469, *p* < 0.001), with the highest ASPR observed in high-income North America, likely linked to diet, obesity, and lifestyle factors (26). Inequality analysis further indicates worsening global health equity in chronic pain burden: the absolute value of the slope index of inequality has increased, reflecting growing disparities across SDI levels, and the CI shows a pronounced pro-rich inequality, with chronic pain burden concentrated in wealthier nations. To better interpret these findings, we now highlight that the observed “pro-rich inequality” challenges the traditional view that chronic pain is mainly a problem of low-income countries, emphasizing its emergence as a major health issue in high-SDI nations and underscoring the need for resource redistribution and preventive strategies in those settings.

The rising burden of chronic pain is multifactorial. In high-income countries, aging and increased longevity have driven up cases of osteoarthritis, spinal disorders, and gout (16). Improved healthcare access in high-SDI regions also contributes to higher diagnosis and reporting rates. Additionally, sedentary behavior, Westernized diets, and elevated stress levels—common in high-income settings—further fuel chronic pain prevalence (27). Conversely, in low-SDI countries, insufficient diagnostic capacity and limited healthcare awareness likely contribute to underreporting, masking the real burden. These inter-regional diagnostic disparities warrant greater global surveillance standardization. Chronic pain thus shows a complex relationship with socioeconomic development. High-SDI countries should enhance prevention and intervention, while low- and middle-SDI regions must improve diagnosis and management to avoid rapid burden escalation. Future global health policies must balance equity and efficiency to manage chronic pain effectively. With chronic pain rising worldwide, high-income nations face aging-driven prevalence, while low-income countries confront dual challenges of limited resources and low awareness, calling for tailored, system-based, and long-term pain strategies (17).

This study analyzed chronic pain distribution by age and sex. Most pain types (except migraine and tension-type headache) become more common with age, disproportionately affecting the older population—likely due to degeneration, chronic inflammation, and multiple health issues (28, 29). Regarding sex differences, gout and pancreatitis were more prevalent in females, whereas conditions such as low back pain, migraine, neck pain, osteoarthritis, and rheumatoid arthritis predominated in males, indicating that sex differences in chronic pain are complex and disease-specific. Literature suggests higher chronic pain prevalence in females may relate to heightened immune responses, estrogen’s role in inflammation modulation, and enhanced neural plasticity (30). Conversely, lifestyle factors more common in males, such as smoking, alcohol use, and heavy physical labor, may drive higher rates of osteoarthritis and spinal disorders (31, 32). Cultural differences in pain expression and healthcare-seeking behaviors between sexes may also bias epidemiological data (31, 32). Addressing these complex pain mechanisms requires innovative strategies, among which platelet-rich plasma has emerged as a promising regenerative therapy for mitigating neuropathic and degenerative pain (33).

Notably, while gout has traditionally been considered more prevalent in males, our study found that in some regions, the number of female patients now exceeds that of males, suggesting that lifestyle changes and aging may be altering the gender burden patterns. The high prevalence of certain musculoskeletal pain conditions in males highlights the need for targeted health protection within the workforce. The accelerated expansion of older populations has emerged as a critical amplifier of chronic pain burden (10). Advanced age stands confirmed as one of the most potent independent risk factors for chronic pain development, with individuals ≥60 years constituting the predominant patient cohort (34). Future pain management strategies should focus on the older population, promoting non-pharmacological interventions such as physical therapy and cognitive behavioral therapy, alongside multidisciplinary treatment models, to alleviate their long-term health and economic burden.

An autoregressive integrated moving average model projections indicate a divergent trend in chronic pian related disease prevalence over the next decade (2022–2032). Specifically, decreasing trends are anticipated for gout, low back pain neck pain, and pancreatitis, whereas rheumatoid arthritis and other musculoskeletal disorders demonstrate upward epidemiological trajectories (35). To provide more interpretive depth, we have expanded the discussion to note that such divergence may reflect differences in modifiable lifestyle factors, improved early intervention programs, and evolving health policy priorities across regions. Lumbago and neck pain remain among the most prevalent musculoskeletal disorders globally, with GBD 2019 estimates documenting 2017 prevalence rates of 36.8 and 18.4%, respectively (36). Nevertheless, temporal analyses indicate a progressive escalation in neck pain prevalence and associated disability burden from 1990 to 2019, with accelerated growth post-2011 (37). Notably, emerging GBD 2021 data suggest potential stabilization of neck pain prevalence in select geographic regions, a phenomenon potentially attributable to enhanced public health initiatives targeting musculoskeletal health, optimized preventive strategies, and advancements in therapeutic approaches (38). These projections underscore the importance of continuous surveillance and adaptive prevention strategies, as the dynamics of chronic pain may shift with social, occupational, and behavioral transitions worldwide.

Gout prevalence, having increased over past decades, may decrease due to lifestyle changes and early intervention. In contrast, rheumatoid arthritis and other musculoskeletal disorders continue to rise (39). Epidemiological modeling estimates 494 million global cases of other musculoskeletal disorders in 2020, with projections forecasting a doubling to 1.06 billion by 2050 (40). These trends underscore the urgent need to strengthen global efforts in preventing, diagnosing, and treating chronic pain–related conditions. For diseases with declining prevalence, sustaining progress requires continued implementation of effective measures; for those increasing, intensified research, new therapies, and heightened public awareness are essential. Policymakers must adapt resource allocation and health strategies in response to evolving disease burdens (41, 42). As chronic pain–related prevalence shifts over the next decade, understanding its drivers is critical to guide targeted interventions that reduce burden and improve population health.

Correlation analysis using the GBD 2021 database revealed links between specific chronic pain conditions and cardiovascular diseases. Gout and osteoarthritis showed strong correlations with non-rheumatic valvular and ischemic heart disease. Rheumatoid arthritis and other musculoskeletal disorders correlated with atrial fibrillation/flutter and endocarditis. In contrast, pancreatitis showed a negative correlation with hypertensive heart disease, while tension-type headache was associated with both hypertensive heart disease and peripheral artery disease of the lower limbs.

The strong positive correlations between inflammatory joint diseases such as gout and rheumatoid arthritis and cardiovascular diseases likely stem from shared inflammatory pathways. Systemic inflammation in rheumatoid arthritis increases risks of atherosclerosis, arrhythmias, and heart failure, while hyperuricemia in gout promotes crystal deposition and elevates cardiovascular risk through oxidative stress and endothelial dysfunction (43, 44). The associations of rheumatoid arthritis and other musculoskeletal disorders with atrial fibrillation and endocarditis may relate to inflammation-driven cardiac remodeling (45, 46). The negative correlation between pancreatitis and hypertensive heart disease was further interpreted as possibly resulting from distinct pathophysiological mechanisms or population distribution differences, rather than a true biological inverse relationship. The inverse relationship of tension-type headache with hypertensive heart disease and peripheral artery disease may be explained by its non-inflammatory nature.

This study highlights the need to include cardiovascular health in chronic pain care. Inflammatory joint diseases warrant prioritized cardiovascular risk management, as anti-inflammatory treatments may reduce events (39). Non-inflammatory pain conditions still require overall health attention. To enhance interpretive clarity, we have added that these findings should be viewed as epidemiological associations reflecting overlapping risk pathways, not direct causation, and that further mechanistic and longitudinal studies are warranted. Future research should clarify chronic pain and cardiovascular disease mechanisms to improve interventions; public health efforts must support integrated, multidisciplinary care (41).

In the Limitations section, we have expanded the discussion to explicitly note that while GBD provides unparalleled global comparability, its aggregation may obscure subnational and demographic disparities. We also acknowledge that cultural biases in pain perception and reporting can influence the apparent prevalence, which should be considered when interpreting global trends (47).

## Conclusion

Chronic pain has emerged as a major and continuously growing global public health concern, characterized by marked regional, age, gender, and socioeconomic disparities. The burden of chronic pain continues to rise in countries with high levels of socio-demographic development, while in low- and middle-SDI countries, it may be underestimated due to potential underdiagnosis. Chronic pain not only compromises individual quality of life but also exacerbates the burden of comorbid chronic diseases such as cardiovascular conditions. Confronting this complex and multidimensional health challenge necessitates the establishment of an integrated prevention and management system, grounded in population-specific approaches and supported by multidisciplinary collaboration. Future efforts should focus on advancing research into the interactions between chronic pain and various chronic diseases, optimizing resource allocation, and promoting policies that prioritize early intervention and health equity, with the ultimate goal of achieving effective global control of the chronic pain burden.

## Data Availability

The datasets presented in this study can be found in online repositories. The names of the repository/repositories and accession number(s) can be found in the article/Supplementary material.

## Footnotes

^1^Institute for Health Metrics and Evaluation. GBD data and tools guide, https://www.healthdata.org/researchanalysis/about-gbd/gbd-data-and-tools-guide (accessed April 15, 2024).
