# Supplementary material for: Global and regional burden, temporal trends, and projections of chronic pain from 1990 to 2032, and its association with cardiovascular diseases: analyses based on global burden of diseases study 2021
Source: Front Public Health. 2025 Nov 14;13:1636949. doi: 10.3389/fpubh.2025.1636949 (PMC12660301; doi:10.3389/fpubh.2025.1636949)
Supplement: Supplementary file 1 [file Supplementary_file_1.pdf]

Supplementary table 1. Selected diagnoses for the evaluation of chronic pain

Category	CIE-10
Gout	M10, M100, M1000, M1001, M10011, M10012, M10019, M1002, M10021, M10022, M10029, M1003, M10031, M10032, M10039, M1004, M10041, M10042, M10049, M1005, M10051, M10052, M10059, M1006, M10061, M10062, M10069, M1007, M10071, M10072, M10079, M1008, M1009, M101, M1010, M1011, M10111, M10112, M10119, M1012, M10121, M10122, M10129, M1013, M10131, M10132, M10139, M1014, M10141, M10142, M10149, M1015, M10151, M10152, M10159, M1016, M10161, M10162, M10169, M1017, M10171, M10172, M10179, M1018, M1019, M102, M1020, M1021, M10211, M10212, M10219, M1022, M10221, M10222, M10229, M1023, M10231, M10232, M10239, M1024, M10241, M10242, M10249, M1025, M10251, M10252, M10259, M1026, M10261, M10262, M10269, M1027, M10271, M10272, M10279, M1028, M1029, M103, M1030, M1031, M10311, M10312, M10319, M1032, M10321, M10322, M10329, M1033, M10331, M10332, M10339, M1034, M10341, M10342, M10349, M1035, M10351, M10352, M10359, M1036, M10361, M10362, M10369, M1037, M10371, M10372, M10379, M1038, M1039, M104, M1040, M1041, M10411, M10412, M10419, M1042, M10421, M10422, M10429, M1043, M10431, M10432, M10439, M1044, M10441,

Category	CIE-10
	M10442, M10449, M1045, M10451, M10452, M10459, M1046, M10461, M10462, M10469, M1047, M10471, M10472, M10479, M1048, M1049, M109
Chronic low back pain and neck pain	M403, M404, M405, M410, M411, M412, M413, M414, M415, M418, M419, M45X, M460, M461, M462, M463, M464, M465, M468, M469, M471, M472, M478, M479, M480, M481, M482, M483, M484, M485, M488, M489, M490, M491, M492, M493, M494, M495, M498, M510, M511, M512, M513, M518, M519, M532, M533, M538, M539, M540, M541, M543, M544, M545, M546, M548, M990, M991, M992, M993, M994, M995, M996, M997, M998, M999
Headache disorders	G43, G430, G4300, G43001, G43009, G4301, G43011, G43019, G431, G4310, G43101, G43109, G4311, G43111, G43119
Cancer-related pain	C000, C001, C002, C003, C004, C005, C006, C008, C009, C01X, C020, C021, C022, C023, C024, C028, C029, C030, C031, C039, C040, C041, C048, C049, C050, C051, C052, C058, C059, C060, C061, C062, C068, C069, C07X, C080, C081, C088, C089, C090, C091, C098, C099, C100, C101, C102, C103, C104, C108, C109, C110, C111, C112, C113, C118, C119, C12X, C130, C131, C132, C138, C139, C140, C142, C148, C150, C151, C152, C153, C154, C155, C158, C159, C160, C161, C162, C163, C164, C165, C166, C168, C169, C170, C171, C172, C173, C178, C179, C180, C181,

Category	CIE-10
	C182, C183, C184, C185, C186, C187, C188, C189, C19X, C20X, C210, C211, C212, C218, C220, C221, C222, C223, C224, C227, C229, C23X, C240, C241, C248, C249, C250, C251, C252, C253, C254, C257, C258, C259, C260, C261, C268, C269, C300, C301, C310, C311, C312, C313, C318, C319, C320, C321, C322, C323, C328, C329, C33X, C340, C341, C342, C343, C348, C349, C37X, C380, C381, C382, C383, C384, C388, C390, C398, C399, C400, C401, C402, C403, C408, C409, C410, C411, C412, C413, C414, C418, C419, C430, C431, C432, C433, C434, C435, C436, C437, C438, C439, C440, C441, C442, C443, C444, C445, C446, C447, C448, C449, C450, C451, C452, C457, C459, C460, C461, C462, C463, C467, C468, C469, C470, C471, C472, C473, C474, C475, C476, C478, C479, C480, C481, C482, C488, C490, C491, C492, C493, C494, C495, C496, C498, C499, C500, C501, C502, C503, C504, C505, C506, C508, C509, C510, C511, C512, C518, C519, C52X, C530, C531, C538, C539, C540, C541, C542, C543, C548, C549, C55X, C56X, C570, C571, C572, C573, C574, C577, C578, C579, C58X, C600, C601, C602, C608, C609, C61X, C620, C621, C629, C630, C631, C632, C637, C638, C639, C64X, C65X, C66X, C670, C671, C672, C673, C674, C675, C676, C677, C678, C679, C680, C681, C688, C689, C690, C691, C692, C693, C694, C695,

Category	CIE-10
	C696, C698, C699, C700, C701, C709, C710, C711, C712, C713, C714, C715, C716, C717, C718, C719, C720, C721, C722, C723, C724, C725, C728, C729, C73X, C740, C741, C749, C750, C751, C752, C753, C754, C755, C758, C759, C760, C761, C762, C763, C764, C765, C767, C768, C770, C771, C772, C773, C774, C775, C778, C779, C780, C781, C782, C783, C784, C785, C786, C787, C788, C790, C791, C792, C793, C794, C795, C796, C797, C798, C80X, C810, C811, C812, C813, C817, C819, C820, C821, C822, C827, C829, C830, C831, C832, C833, C834, C835, C836, C837, C838, C839, C840, C841, C842, C843, C844, C845, C850, C851, C857, C859, C880, C881, C882, C883, C887, C889, C900, C901, C902, C910, C911, C912, C913, C914, C915, C917, C919, C920, C921, C922, C923, C924, C925, C927, C929, C930, C931, C932, C937, C939, C940, C941, C942, C943, C944, C945, C947, C950, C951, C952, C957, C959, C960, C961, C962, C963, C967, C969, C97X, G731, C964, C968, C928, C799, C800, C809, C814, C823, C825, C846, C847, C848, C849, C852, C860, C862, C863, C864, C866, C884, C903, C918, C946, C965, C966
Osteoarthritis	M150, M151, M152, M153, M154, M158, M159, M160, M161, M162, M163, M164, M165, M166, M167, M169, M170, M171, M172, M173, M174, M175, M179, M180, M181, M182, M183, M184, M185, M189,

Category	CIE-10
	M190, M191, M192, M198, M199
Other musculoskeletal conditions	M050, M051, M052, M053, M058, M059, M060, M061, M062, M063, M064, M068, M069, M080, M081, M082, M083, M084, M088, M089, M139, M255, M353, M438, M754, M774, M791, M796
Pancreatitis	K861
Rheumatoid arthritis	M05
herpes zoster	B02, B0239

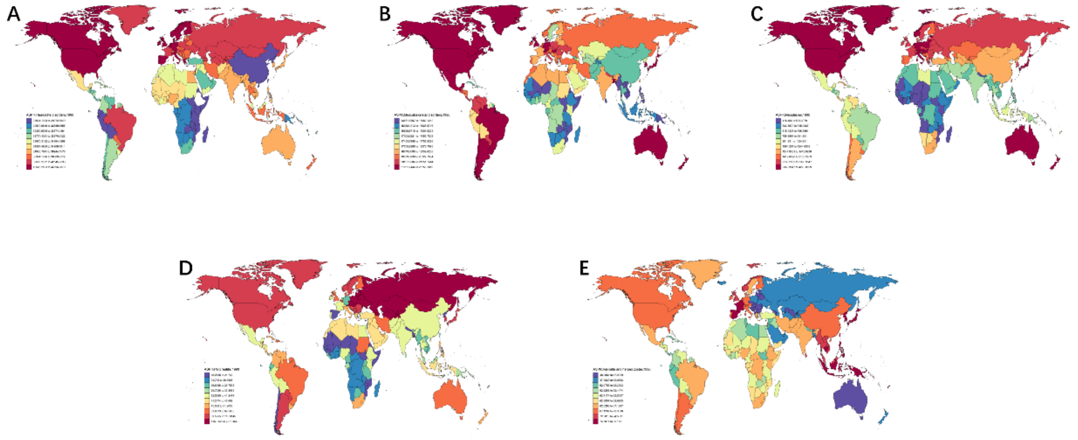

Supplementary Figure 1 Distribution of ASPR for headache disorders (A), musculoskeletal disorders (B), neoplasms (C), pancreatitis (D) and herpes zoster (E) in 204 countries (regions) worldwide in 1990.

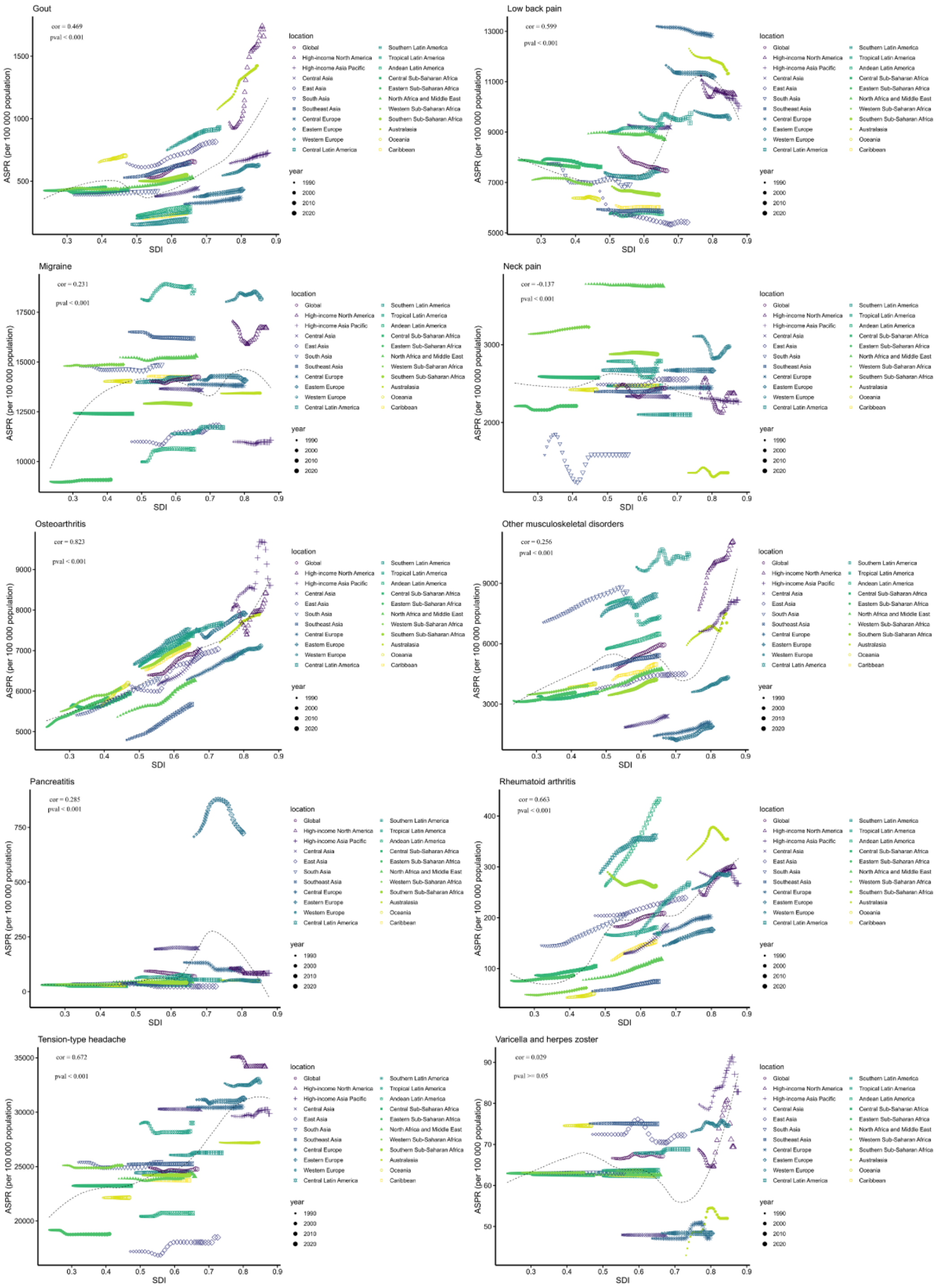

Supplementary figure 2 Association between ASPR and SDI for different chronic pains

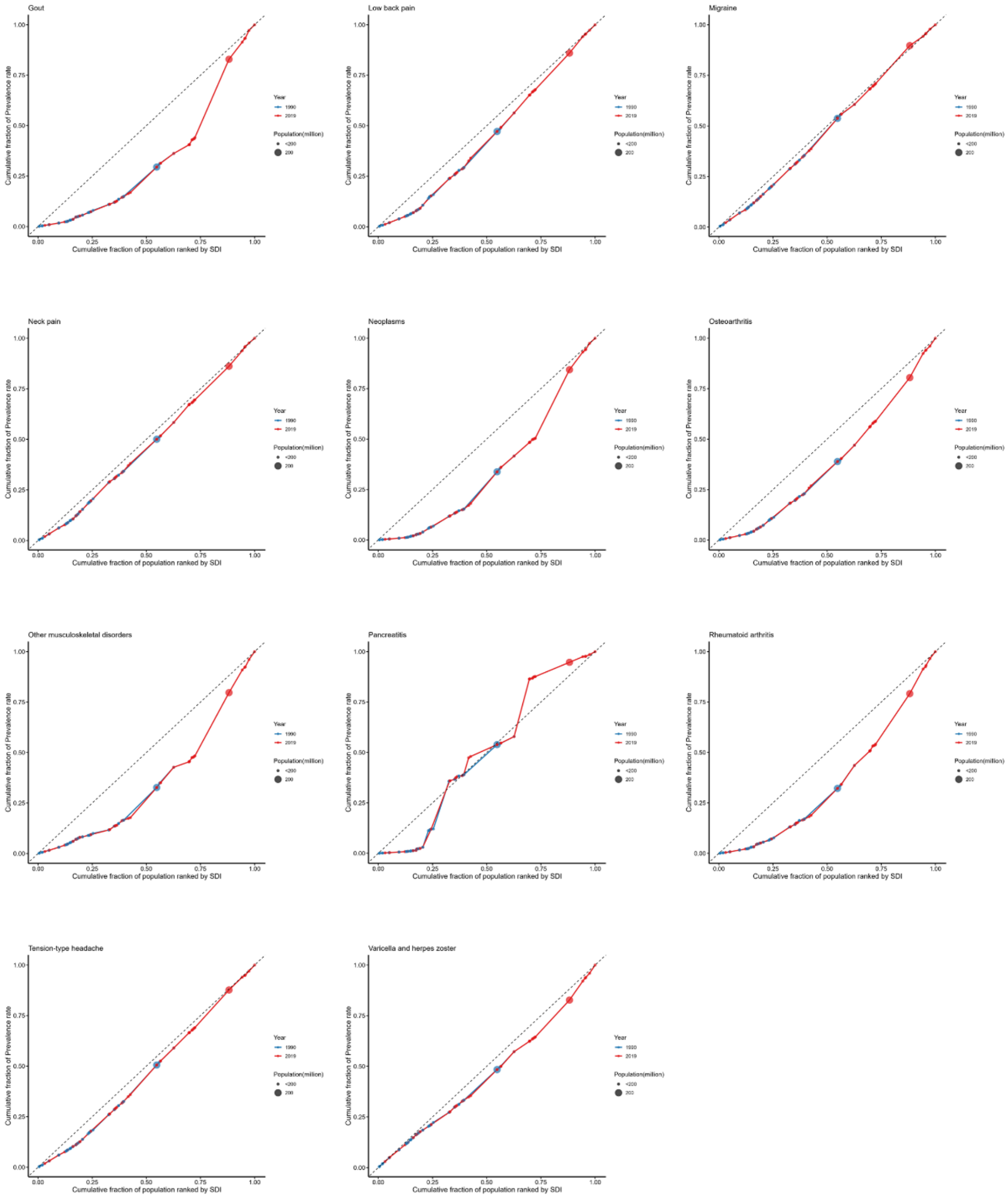

Supplementary figure 3 Concentration index of chronic pain prevalence
